# Clinical outcomes for pleomorphic xanthoastrocytoma patients: an institutional experience

**DOI:** 10.21203/rs.3.rs-2535551/v1

**Published:** 2023-02-03

**Authors:** Jared Sullivan, James Chandler, Maciej Lesniak, Matthew Tate, Adam Sonabend, John Kalapurakal, Craig Horbinski, Rimas Lukas, Priya Kumthekar, Sean Sachdev

**Affiliations:** Northwestern University; Northwestern University Robert H. Lurie Comprehensive Cancer Center; Northwestern University Robert H. Lurie Comprehensive Cancer Center; Northwestern University Robert H. Lurie Comprehensive Cancer Center; Northwestern University Robert H. Lurie Comprehensive Cancer Center; Northwestern University Robert H. Lurie Comprehensive Cancer Center; Northwestern University Robert H. Lurie Comprehensive Cancer Center; Northwestern University Robert H. Lurie Comprehensive Cancer Center; Northwestern University Robert H. Lurie Comprehensive Cancer Center; Northwestern University Robert H. Lurie Comprehensive Cancer Center

**Keywords:** PXA, anaplastic, surgery, radiotherapy, BRAF, V600E mutation

## Abstract

**Purpose:**

Report our institutional experience with pleomorphic xanthoastrocytoma (PXA) to contribute to limited data on optimal management.

**Methods:**

Patients with pathologically confirmed PXA treated at our institution between 1990 and 2019 were identified. Demographic information, tumor grade, treatment variables, and clinical outcomes were collected from patient charts. Kaplan-Meier estimates were used to summarize two primary outcome measurements: progression-free survival (PFS) and overall survival (OS). Outcomes were stratified by tumor grade and extent of resection. Cox regression and log-rank testing were performed.

**Results:**

We identified 17 patients with pathologically confirmed PXA. Two patients were excluded due to incomplete treatment information or < 6m of follow-up; 15 patients were analyzed (median follow-up 4.4y). Six patients had grade 2 PXA and 9 had grade 3 anaplastic PXA. The 2-year and 5-year PFS for the cohort was 57% and 33%, respectively; 2-year and 5-year OS was 93% and 75%, respectively. Patients with grade 2 tumors exhibited superior PFS compared to those with grade 3 tumors (2-year PFS: 100% vs. 28%, 5-year PFS: 60% vs. 14%), hazard ratio, 5.09 (95% CI:1.06–24.50), p = 0.02. Undergoing a GTR also yielded improved outcomes (hazard ratio: 0.38, p = 0.15). All but one (89%) of the grade 3 patients underwent RT.

**Conclusion:**

The poor survival of the cohort, especially with grade 3 tumors, suggests the need for more aggressive treatment, including maximal resection followed by intensive adjuvant therapy. Better prognostics of tumor recurrence are needed to guide the use of adjuvant therapy.

## Introduction

Pleomorphic xanthoastrocytoma (PXA) is a rare astrocytic tumor that mainly affects children and young adults, with a mean age of diagnosis of 29 [1]. First described by Kepes et al. in 1979 [2], PXA carries a generally favorable prognosis with a 5-year overall survival (OS) rate of > 75% and progression-free survival (PFS) rate of > 60% [1]. Most PXA cases are WHO grade 2, which can often be definitively treated with surgery alone. Anaplastic grade 3 variants (aPXA) have higher potential for CSF spread and a higher likelihood of recurrence after initial resection, leading to poorer PFS and OS [1, 3–5].

Given the rarity of the tumor, there is a lack of data to guide appropriate treatment. Based on the results of several retrospective single- and multi-institution studies correlating gross total resection (GTR) with longer PFS [3, 5–8], GTR, if feasible and safe, has often been considered the mainstay of treatment. This aligns with the contemporary classification of PXA as a circumscribed astrocytic glioma [9]. The role of adjuvant and salvage radiotherapy and chemotherapy in the treatment of PXA remains uncertain. Despite the paucity of data, several authors suggest a possible benefit of radiotherapy in the setting of residual or recurrent disease, as well as in more aggressive cases of aPXA [3, 5, 7, 8, 10–15]. The use of traditional alkylating chemotherapy agents like temozolomide (TMZ) has shown limited efficacy against PXA [5, 7, 13, 16].

Molecular characterization of PXA tumors has identified a common missense mutation (V600E) in the BRAF kinase gene that may be a driving mutation, present in roughly two-thirds of PXA tumors [17], making BRAF molecular profiling an important diagnostic tool for PXA. BRAF V600E mutation status also appears to have prognostic value, with longer OS noted for BRAF V6000E-mutated PXAs [5]. This molecular finding is more frequently observed in WHO grade 2 PXA than in WHO grade 3 [1, 18]. More recently, BRAF inhibition (BRAFi), either alone or in combination with MEK inhibition (MEKi), has shown promise in patients harboring the V600E mutation in several case reports, case series, and most notably, the VE-BASKET phase II clinical trial of BRAFi agent, vemurafenib, and the ROAR phase II trial of the BRAFi agent, dabrafenib, and the MEKi agent, trametinib [19–26].

In the present study, we report our institutional experience with PXA along with an analysis of the demographic, prognostic, and treatment-related variables. We aim to better understand optimal clinical management of patients with this rare brain tumor type.

## Methods

### Patient identification

We identified patients treated for PXA at our institution between 1990 and 2019. Patients for whom histopathologic analysis of tumor tissue showed PXA as the most likely diagnosis were included for further analysis.

### Data collection

Clinical, pathologic, and treatment characteristics were collected from patient charts. Tumor grade was determined based on histopathologic analysis. Extent of resection was defined by the surgeon’s impression and radiographic assessment. Lesions that were completely resected were classified as gross total resection (GTR). Lesions that were incompletely resected, “debulked,” or with unknown extent of resection were classified as subtotal resection (STR). Date of tumor recurrence was determined by imaging concern for recurrent disease. Radiotherapy administered up to 4 months after initial surgery was considered adjuvant; when administered after recurrence, it was considered as salvage treatment.

### Statistical analysis

The primary outcome measurements in this study were PFS and OS. PFS was defined as time from pathologic diagnosis to tumor progression or recurrence, or time to death or last known radiologic follow-up. OS was defined as time from pathologic diagnosis to death or last known clinical follow-up. Kaplan-Meier estimates were used to summarize PFS and OS. Outcomes were analyzed according to important pathologic and treatment variables including tumor grade, BRAF V600E mutational status, extent of resection, and use of adjuvant therapy. Cox proportional hazards regression was utilized to assess the independent effects of these variables. A p < 0.05 was considered statistically significant.

## Results

### Patient & tumor characteristics

We identified seventeen adult patients with pathologically confirmed PXA. Two patients were excluded due to incomplete treatment information or insufficient follow-up (< 6 months). Fifteen patients were analyzed (Table 1). The median age at diagnosis was 35 years (range: 10–71), with five females (33%) and ten males (67%). Of the fifteen included patients, six had grade 2 PXA (40%), and nine had grade 3 anaplastic PXA (60%). Seven patients were BRAF V600E-positive (47%), one was BRAF V600E-negative, and seven were not tested.

### Treatment characteristics

All patients underwent initial surgical resection, with seven GTR (47%). In total, eight patients received adjuvant RT (53%), three received salvage RT (20%), and four did not receive any RT (27%). Eight of the nine (89%) grade 3 PXA patients and three of the six grade 2 PXA patients (50%) received RT. Eight patients received concurrent TMZ (72%). Four of the seven BRAF V600E-positive patients (57%) received targeted BRAFi therapy.

### Survival outcomes

**Figure 1:**
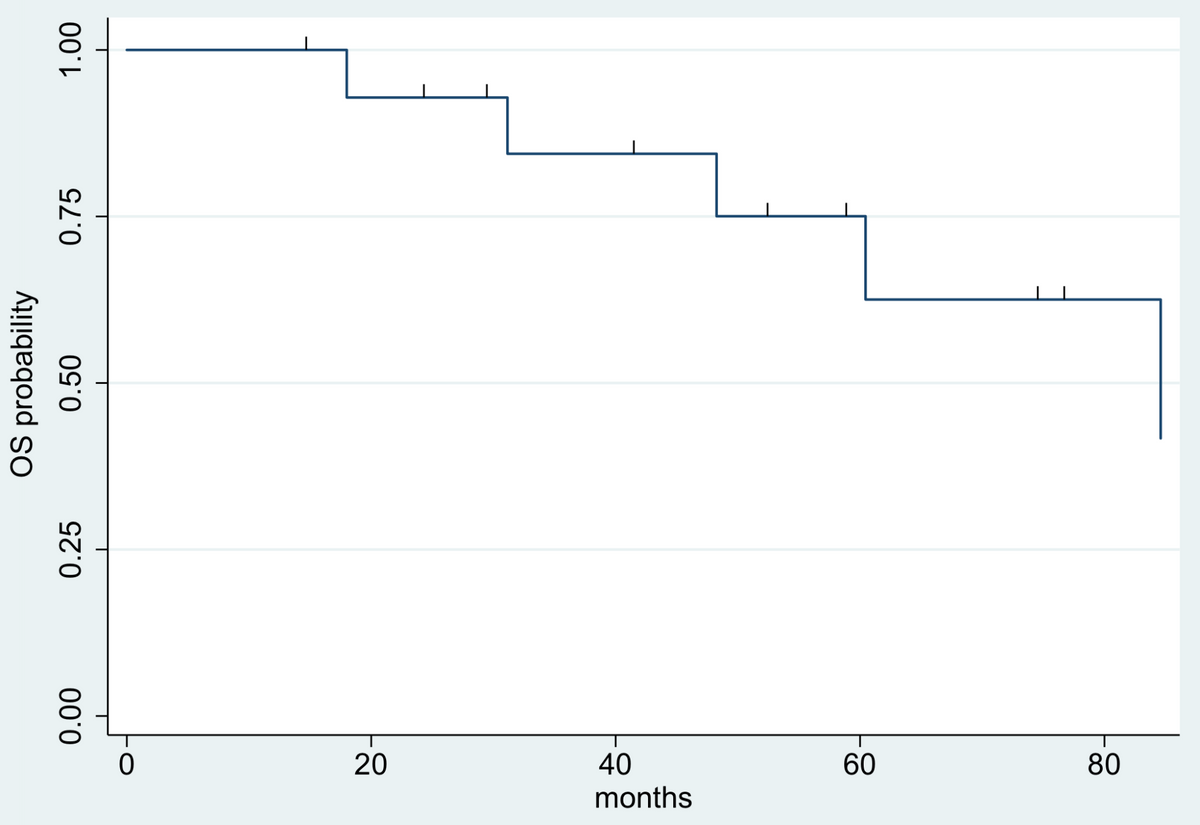
Kaplan-Meier survival curve showing overall survival across the full cohort.

**Figure 2:**
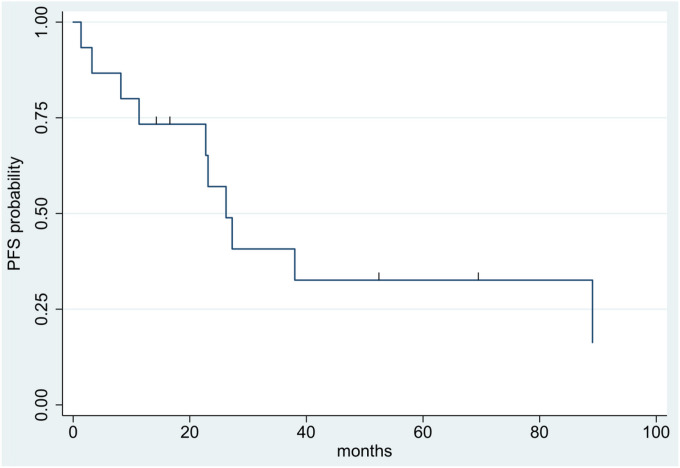
Kaplan-Meier survival curve showing progression-free survival across the full cohort.

Median follow-up for all included patients was 4.4 years (range: 1.2–23.5 years). Ten patients experienced tumor progression or recurrence–including two of the six grade 2 patients and eight of the nine grade 3 patients. Median time to progression/recurrence was 1.9 years. The two-year and five-year OS for the entire cohort was 93% and 75%, respectively (Fig. 1). The two-year and five-year PFS was 57% and 33%, respectively (Fig. 2).

**Figure 3:**
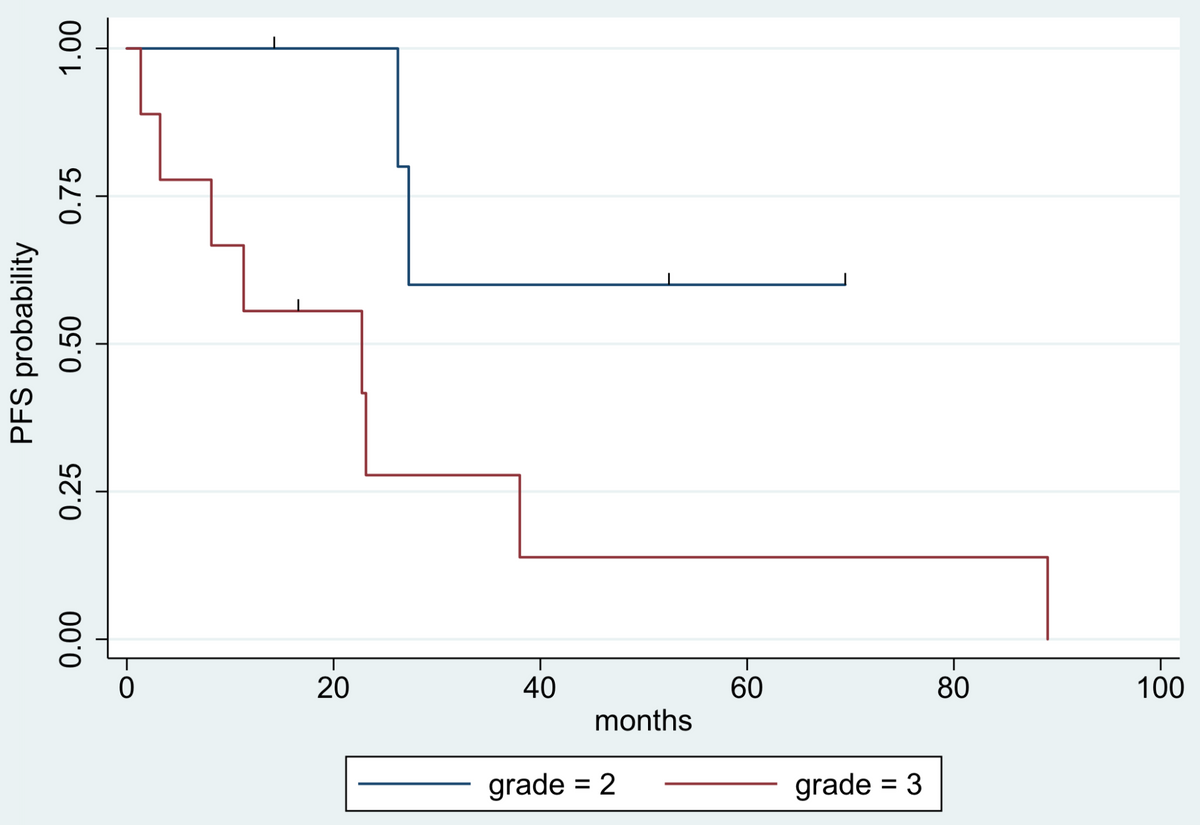
Kaplan-Meier survival curves showing progression-free survival stratified by tumor grade. Blue line = grade 2, red line = grade 3.

**Figure 4:**
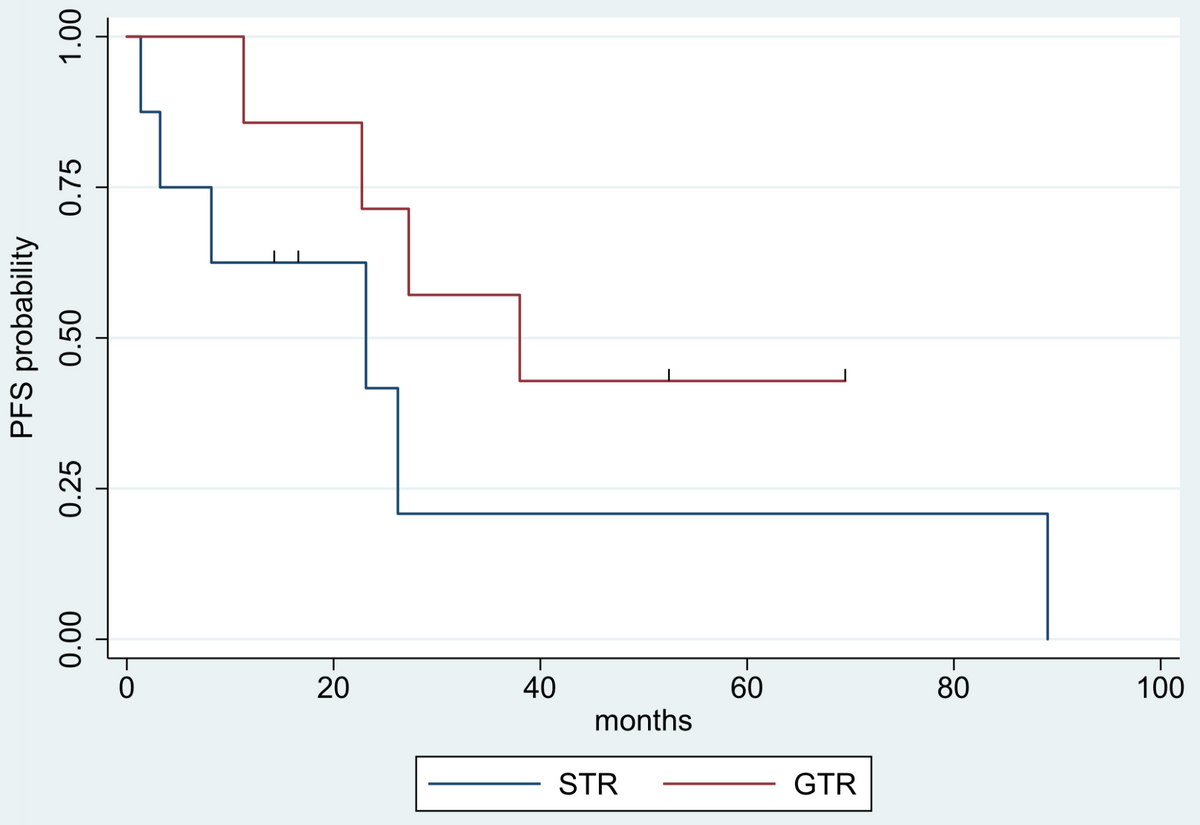
Kaplan-Meier survival curves showing progression-free survival stratified by extent of resection. Blue line = STR, red line = GTR.

Patients with grade 2 tumors exhibited superior PFS compared to those with grade 3 tumors (two-year PFS: 100% vs. 28%; five-year PFS: 60% vs. 14%) (Fig. 3); hazard ratio for grade 3 vs. grade 2 tumors was 5.09 (95% CI: 1.06–24.50, p = 0.02). Gross total resection yielded improved PFS compared to STR (two-year PFS: 71% vs. 42%; five-year PFS: 43% vs. 21%) (Fig. 4), but this result was not statistically significant (hazard ratio: 0.38, p = 0.15). BRAF V600E mutational status (positive vs. negative and not tested) and the use of adjuvant therapies were not statistically significant predictors of outcomes.

## Discussion

PXA can be a recalcitrant and malignant tumor, especially in the setting of the grade 3 anaplastic histology [4, 5]. Maximal safe surgical resection may offer adequate disease control without the need for further adjuvant therapy [1]. However, there is a wide range of clinical outcomes including aggressive behavior and/or worse survival [1, 27]. The role of adjuvant radiation and/or systemic therapy following maximal resection in PXA patients remains unclear.

### Tumor grade

The prognostic value of the histologic grade of PXA tumors has been well documented across several studies [3, 5, 14, 27], with consistently longer PFS observed in patients with grade 2 tumors. The findings of our current series are in agreement with these past findings as a significant more favorable PFS was noted in patients with grade 2 vs. grade 3 tumors (Fig. 3). The difference in prognosis based on histologic grade suggests that a more aggressive adjuvant treatment strategy may be necessary for aPXA patients.

### Extent of resection

The majority of past studies have found that extent of resection has significant prognostic significance in patients with PXA, impacting PFS more profoundly than OS [4, 6–8, 28]. While our study did not find a significant difference in survival outcomes based on extent of resection, the numerical trend favored a survival advantage in patients undergoing GTR (Fig. 4), a finding consistent with existing literature. The lack of significance can likely be partially explained by our relatively small sample size and corresponding lack of statistical power. Our series therefore supports the current clinical management of PXA with initial maximal resection. This aligns with the current conceptualization of PXA as a circumscribed astrocytic tumor, implying potential for cure with surgical resection.

### Radiation therapy

The role of radiation therapy in the management of PXA patients has been limited by lack of data, with no previous studies (to our knowledge) finding a significant survival benefit in patients managed with adjuvant or salvage RT. However, many authors have suggested that the use of adjuvant/salvage RT should be considered in cases of recurrent, residual, or metastatic disease [6–8, 14, 29, 30]. This recommendation is supported by several reports showing good disease control in aPXA patients treated with adjuvant RT +/− chemotherapy [10–12, 16]. The patients in our cohort indeed underwent more frequent RT in the setting of characteristics such as anaplastic histology or subtotal resection.

### Chemotherapy & BRAF inhibition

Traditional chemotherapy is considered to be minimally effective in the treatment of PXA [1, 4, 13, 16]. However, BRAF inhibiting agents like vemurafenib, dabrafenib, and encorafenib are an emerging treatment option for the PXA patients harboring the V600E mutation [5]. Several studies have noted the positive prognostic significance of the V600E BRAF mutation [1, 5, 17, 24], which is typically seen in less aggressive tumors [17]. Case reports [19, 21, 22], and more recently longitudinal clinical trials [24, 25], have shown survival benefits for V600E-positive PXA patients treated with BRAF inhibitors. As BRAFi therapy is more consistently employed in the management of V600E-positive PXAs, more robust data on its efficacy may be generated ahead.

### Angiomatous PXA

We make note of one patient in the present series who had angiomatous features on pathologic diagnosis, constituting a very rare PXA variant. To our knowledge, only seven cases of angiomatous PXA have been reported in the literature, without a single reported recurrence, underscoring the indolent nature of this variant [31–36]. The one angiomatous PXA patient in our series, a 45-year-old male with a grade 2 tumor, underwent GTR without any adjuvant therapy. He did not experience tumor progression or recurrence for a 22.5 year follow-up, the longest reported follow-up in the literature for angiomatous PXA. This single case adds to the limited body of literature on this variant, providing further evidence of the indolent behavior of angiomatous PXA.

### Limitations

Our study has several limitations. First, it is a single-institution, retrospective study. Given the rarity of PXA, this is a difficult limitation to overcome, yet our series is still one of the largest reported in the literature. Next, perhaps as a tertiary care center, we had a higher proportion of grade 3 aPXA patients (60%) compared to the 25–30% reported in the wider population [37]. While this may limit the generalizability of our series, it provides important outcomes data on the more aggressive variants of aPXA. Finally, molecular profiling of tumors has only recently become common practice meaning only the patients in our cohort diagnosed later in the follow-up period have a recorded BRAF V600E mutational status, precluding robust analysis of the prognostic significance of BRAF mutational status.

## Conclusion

Our findings align with previous reports of the aggressive nature of grade 3 anaplastic PXA compared to grade 2 PXA. The poor survival of the cohort, especially with grade 3 tumors, suggests the need for more aggressive treatment, including maximal resection followed by intensive adjuvant therapy and when possible, BRAF inhibitor therapy for V600E-positive PXA. Better prognosticators of tumor recurrence are needed to guide the use of adjuvant therapy.

## Figures and Tables

**Table 1:** Characteristics of patients.

Variable	Number (%)
**Age (years)**	
Median	35
Range	10–71
**Sex**	
Male	10 (67)
Female	5 (33)
**Grade**	
2	6 (40)
3	9 (60)
**BRAF mutation**	
Yes	7 (47)
No	1 (7)
Not tested	7 (47)
**Gross Total Resection**	
Yes	7 (47)
No	6 (40)
Unknown	2 (13)
**Radiation Therapy**	
Adjuvant	8 (53)
Salvage	3 (20)
No	4 (27)
**Radiation Therapy (n = 11)**	
SRS	1 (10)
EBRT	10 (90)
Median Dose (Gy)	60.0
Concurrent Temozolomide	8 (72)

Abbreviations: SRS = stereotactic radiosurgery, EBRT = external beam radiation therapy
